# Causal effect of education on bone mineral density: A Mendelian randomization study

**DOI:** 10.1097/MD.0000000000037435

**Published:** 2024-03-15

**Authors:** Xiaoqing Mou, Mingqi Sun, Xiaojun Chen

**Affiliations:** a Department of Radiology, The Affiliated Hospital, Southwest Medical University, Luzhou, Sichuan, China; b Department of Orthopaedic Trauma, The Second Affiliated Hospital of Inner Mongolia Medical University, Huhhot, Inner Mongolia, China; c Department of Orthopedics, The Affiliated Traditional Chinese Medicine Hospital, Southwest Medical University, Luzhou, Sichuan, China.

**Keywords:** BMD, education, Mendelian randomization study, osteoporosis

## Abstract

Education level may have some association with the incidence of osteoporosis, but it is elusive if this association is causal. This two-sample Mendelian randomization analysis focused on the causal effect of education level on femoral neck bone mineral density (FN-BMD), forearm BMD, lumbar spine BMD, and heel BMD. Twelve single nucleotide polymorphisms were used as instrumental variables. The results suggested that high education level was associated with improved FN-BMD (beta-estimate: 0.406, 95% confidence interval: 0.061 to 0.751, standard error: 0.176, *P*-value = .021). There were null association between education and other sites of bone mineral density. Our results found the causal effect of high education level on improved FN-BMD, and improved educational attainment may be beneficial to prevent osteoporosis.

## 1. Introduction

The ratio of people aged more than 65 years to those aged 15 to 64 years is estimated to triple globally by 2100.^[1]^ As the aging of society, disordered musculoskeletal conditions may lead to severe pain and physical disability.^[2]^ Especially, osteoporosis is one common, aging-related disease characterized by decreased bone mineral density (BMD) and increased risk of fracture.^[3–6]^ The treatment of osteoporosis is still a big challenge and serious public health problem.^[7–9]^

Many studies documented that educational level affected the incidence of some chronic diseases such as obesity, diabetes, and cancers.^[10,11]^ A cross-sectional study was conducted to estimate the BMD of women born in Southeast Asia who then lived in Chicago, Illinois, and the results revealed that a high education level had a strong association with improved BMD,^[12]^ but conflicting findings were noted between educational level and osteoporosis in other studies.^[13–15]^ In order to prevent reverse causation and potential confounding factors, two-sample Mendelian randomization (MR) study is developed to establish the causal association between exposure phenotype and outcome phenotype by using the summary genome-wide association studies (GWAS) statistics and instrumental variables.^[16–19]^

GWASs have demonstrated that BMD is a highly polygenic trait.^[20–23]^ In this study, single nucleotide polymorphisms (SNPs) strongly associated with educational attainment are used as instrumental variables. This two-sample MR study aims to explore the causal effect of education level on femoral neck BMD (FN-BMD), forearm BMD (FA-BMD), lumbar spine BMD (LS-BMD) and heel BMD (HE-BMD).

## 2. Methods

### 2.1. Data on education

A large GWAS meta-analysis of educational attainment involved 293,723 people of European descent. Educational attainment was defined by whether the participant attained a given level of schooling based on the International Standard Classification of Education 1997 classification scale. Then, SNPs with the GWAS threshold of statistical significance (*P* < 5*10^−8^) were identified to have robust association with educational attainment.^[24]^

### 2.2. Data on BMD

Osteoporotic fractures commonly occurred in the skeletal sites including femoral neck, forearm, lumbar spine, and heel.^[25,26]^ A large meta-analysis was conducted among 53,236 individuals of European ancestry and aimed to identify genetic variants associated with FN-BMD, FA-BMD, and LS-BMD. Each SNP was tested after adjusting for sex, age, age^2^ and weight.^[25]^ In addition, the GWAS summary data related to HE-BMD were obtained from 426,824 individuals of European ancestry after adjusting for age, sex, and genotyping.^[3]^

### 2.3. Instrumental variable selection

The instrumental variables were selected according to the following 3 assumptions: (i) instrumental SNPs were robustly associated with the education based on the GWAS threshold of *P* < 5 × 10^-8^; (ii) instrumental variables affected outcomes only through their effect on education level and not through any alternative causal pathway; and (iii) instrumental SNPs were independent of any confounders.^[27]^ For SNPs that were unavailable in the outcome dataset, proxy SNPs in LD (r^2^ > 0.8) were used as instrumental variables, but one genetic SNP would be excluded if its proxy could not be searched from the outcome GWAS. In one MR study, SNPs in strong LD may produce some bias, and thus SNPs should be not in linkage disequilibrium (LD). SNP with high LD (r^2^ ≥ 0.001) would be removed.

### 2.4. Statistical analyses

To study MR estimates of educational attainment on FN-BMD, FA-BMD, LS-BMD, and HE-BMD, we conducted the inverse variance weighted (IVW) meta-analysis of the Wald ratio for individual SNPs. The weighted median and MR-Egger regression methods were also applied to perform the sensitivity analysis. The strength of each instrument SNP was measured by calculating the F-statistic using the following formula: F = R^2^(N−2)/(1−R^2^), where R^2^ was the proportion of the education variability and N was the sample size.^[28]^ The directional pleiotropy was assessed via the intercept obtained from the MR-Egger analysis.^[29]^

The ethical approval was not necessary, because this MR study was conducted based on the GWAS summary data of published studies. The ethical approval for each study included in the MR study can be found in the original publications (including informed consent from each participant). All tests were two-tailed, and differences with *P* < .05 were considered statistically significant. All of these analyses were conducted in R V.4.0.4 by using the R packages of ‘MendelianRandomization’^[30]^ and “TwoSampleMR.”^[31]^

## 3. Results

Twelve SNPs (rs301800, rs11210860, rs34305371, rs1008078, rs11588857, rs1777827, rs2992632, rs76076331, rs11689269, rs11690172, rs2457660, rs10496091) were selected as the instrumental variables (Table 1). The beta-estimates of each independent SNPs associated with education and outcomes measurements (FN-BMD, FA-BMD, LS-BMD, and HE-BMD) were provided in Table 2, and no SNP was removed due to high LD.

**Table 1:** Summary information of the 12 SNPs for Mendelian randomization analyses from the GWAS meta-analysis.

SNPs	Chr	Position	Allele 1	Frequency allele 1	Effect size	SE	*P*-value	Samples size	R^2^	F-statistic
rs301800	1	8490603	T	0.1807	0.01911185	1.794E−08	.0034	293,723	1.08E−04	32
rs11210860	1	43982527	A	0.3721	0.01710233	2.359E−10	.0027	293723	1.37E−04	40
rs34305371	1	72733610	A	0.0939c	0.03546801	3.762E−14	.005	293723	2.14E−04	63
rs1008078	1	91189731	T	0.4057	−0.0164957	6.005E−10	.0026	293723	1.31E−04	39
rs11588857	1	204587047	A	0.2115	0.01984361	5.272E−10	.0032	293723	1.31E−04	39
rs1777827	1	211613114	A	0.5942	0.01502804	1.547E−08	.0027	293723	1.09E−04	32
rs2992632	1	243503764	A	0.7177	0.01675647	8.227E−09	.0029	293723	1.14E−04	33
rs76076331	2	10977585	T	0.1463	0.0204809	3.632E−08	.0036	293723	1.05E−04	31
rs11689269	2	15621917	C	0.3346	0.01577685	1.283E−08	.0028	293723	1.11E−04	33
rs11690172	2	57387094	A	0.5903	0.01489159	1.994E−08	.0027	293723	1.07E−04	32
rs2457660	2	60757419	T	0.6354	−0.01682802	7.107E−10	.0028	293723	1.31E−04	39
rs10496091	2	61482261	A	0.2902	−0.01782626	5.615E−10	.0029	293723	1.31E−04	38

**Table 2:** Summary statistics of the genetic instruments of education with different phenotypes.

SNPs	Beta	SE	Beta	SE
	**Education**		**FN-BMD**	
rs301800	0.019111853	0.0034	0.014618	0.009739
rs11210860	0.017102334	0.0027	0.008337	0.007788
rs34305371	0.035468014	0.005	0.022214	0.013248
rs1008078	−0.016495697	0.0026	−0.000474	0.007677
rs1777827	0.01502804	0.0027	−0.00236	0.007657
rs2992632	0.016756467	0.0029	0.018047	0.008312
rs11689269	0.015776847	0.0028	−0.002428	0.008026
			**FA-BMD**	
rs301800	0.019111853	0.0034	0.019201	0.020404
rs11210860	0.017102334	0.0027	0.011222	0.016369
rs34305371	0.035468014	0.005	0.0078	0.026578
rs1008078	−0.016495697	0.0026	−0.003892	0.01571
rs11588857	0.019843614	0.0032	−0.007333	0.018913
rs1777827	0.01502804	0.0027	0.002948	0.01609
rs2992632	0.016756467	0.0029	−0.012144	0.017286
rs76076331	0.020480903	0.0036	−0.030386	0.022883
rs11689269	0.015776847	0.0028	0.002264	0.016959
rs11690172	0.014891586	0.0027	0.020893	0.016269
rs2457660	−0.016828016	0.0028	−0.011393	0.015803
rs10496091	−0.017826263	0.0029	−0.019385	0.017271
			**LS-BMD**	
rs301800	0.019111853	0.0034	0.030694	0.011341
rs11210860	0.017102334	0.0027	−0.002349	0.009091
rs34305371	0.035468014	0.005	−0.000976	0.015432
rs1008078	−0.016495697	0.0026	0.004887	0.008914
rs1777827	0.01502804	0.0027	0.011365	0.008923
rs2992632	0.016756467	0.0029	0.004959	0.009714
rs11689269	0.015776847	0.0028	0.015661	0.009369
			**HE-BMD**	
rs301800	0.019111853	0.0034	0.0200508	0.00241906
rs11210860	0.017102334	0.0027	0.00249778	0.00188731
rs34305371	0.035468014	0.005	−0.0147257	0.00302308
rs1008078	−0.016495697	0.0026	−0.00321085	0.0018774
rs11588857	0.019843614	0.0032	−0.00164935	0.00224865
rs1777827	0.01502804	0.0027	−0.00123398	0.00187357
rs2992632	0.016756467	0.0029	−0.00567569	0.00203533

### 3.1. Causal effect of education on FN-BMD

**Figure 1.**
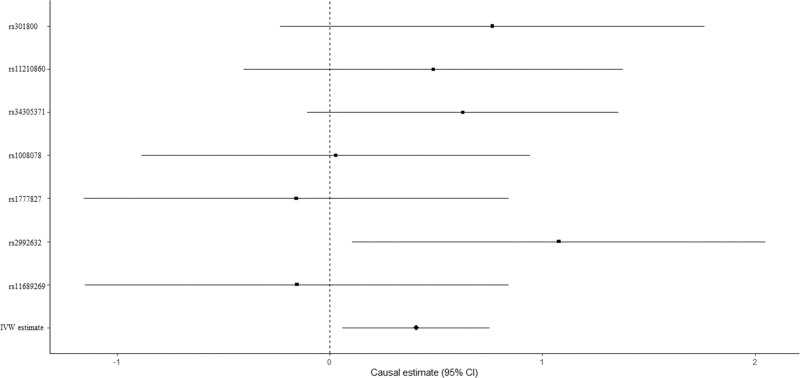
Mendelian randomization association between education and FN-BMD. FN-BMD = femoral neck bone mineral density.

We evaluated the causal effect of education on FN-BMD in the MR analysis. High education level was significantly associated with improved FN-BMD (beta-estimate: 0.406, 95% confidence interval [CI]: 0.061 to 0.751, standard error [SE]: 0.176, *P*-value = .021, Table 3) in the IVW analysis. There was no evidence of heterogeneity according to Cochran Q (Q-value = 5.795, P-heterogeneity = 0.447, I^2^ = 0%). Furthermore, this positive result was also supported by weighted-median analysis (beta-estimate: 0.519, 95% CI: 0.062 to 0.976, SE: 0.233, *P*-value = .026, Table 3). MR association between education and FN-BMD was presented in Figure 1.

**Table 3:** Mendelian randomization estimates of education on outcomes.

Variables	IVW						Weighted median				MR-Egger							
	Estimate	SE	95% CI	*P*-value	Q value	Heterogeneity P value	Estimate	SE	95% CI	*P*-value	Estimate	SE	95% CI	*P*-value	Intercept	SE	95% CI	Pleiotropy P value
FN-BMD	0.406	0.176	0.061,0.751	.021	5.795	.447	0.519	0.233	0.062,0.976	.026	1.140	0.700	−0.231,2.512	.103	−0.014	0.013	−0.039,0.011	0.278
FA-BMD	0.27	0.283	−0.285,0.825	.340	6.480	.840	0.228	0.370	−0.496,0.953	.537	-0.431	1.333	−3.044,2.182	.746	0.013	0.024	−0.034,0.060	0.590
LS-BMD	0.369	0.253	−0.128,0.866	.145	9.143	.166	0.150	0.273	−0.385,0.684	.583	-0.267	1.062	−2.350,1.815	.801	0.012	0.019	−0.026,0.050	0.536
HE-BMD	0.001	0.176	−0.344,0.346	.995	105.854	<.0001	-0.083	0.077	−0.234,0.069	.285	-0.655	0.684	−1.997,0.686	.338	0.013	0.013	−0.013,0.038	0.321

### 3.2. Causal effect of education on FA-BMD and LS-BMD

**Figure 2.**
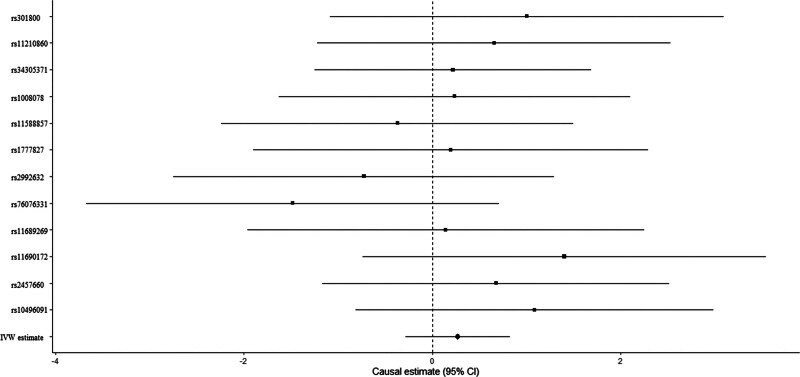
Mendelian randomization association between education and FA-BMD. FA-BMD = forearm bone mineral density.

**Figure 3.**
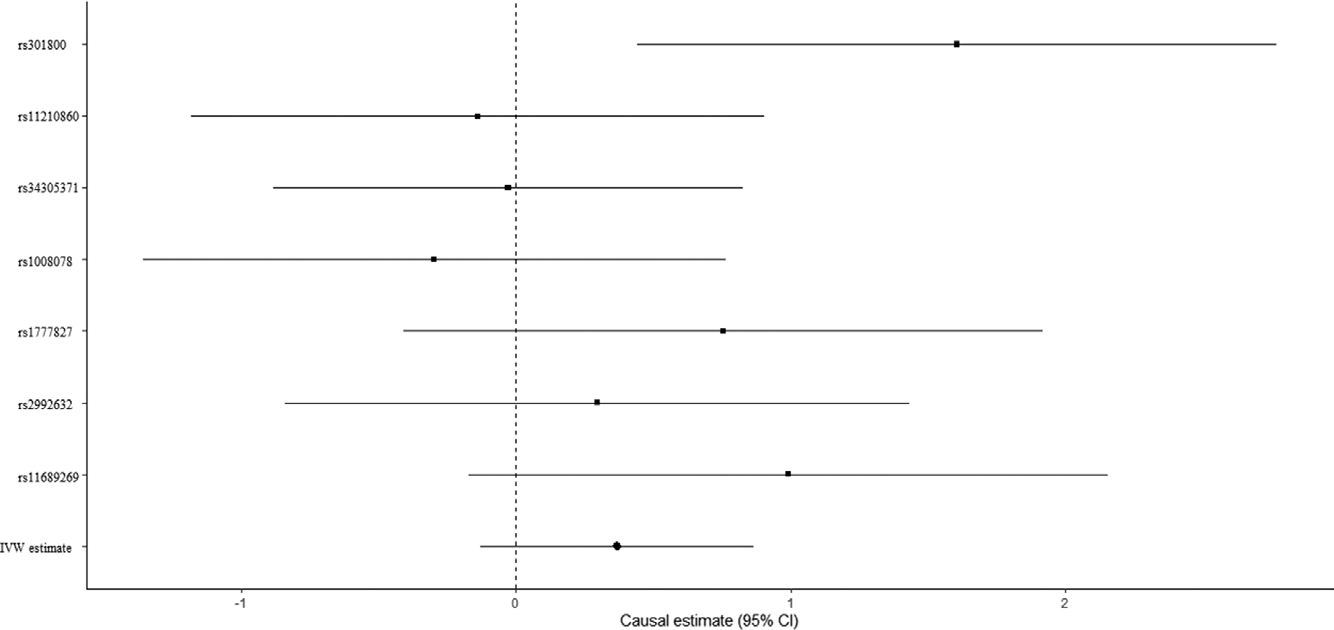
Mendelian randomization association between education and LS-BMD. LS-BMD = lumbar spine bone mineral density.

Education level showed null association with FA-BMD in the IVW (beta-estimate: 0.270 95% CI: −0.285 to 0.825, SE: 0.283, *P*-value = .340) or weighted-median analyses (beta-estimate: 0.228, 95% CI: −0.496 to 0.953, SE: 0.370, *P*-value = .537, Table 3). No evidence of heterogeneity was observed based on Cochran Q (Q-value = 6.4795, P-heterogeneity = 0.8395, I^2^ = 0%). Consistently, there was also no relationship between education and LS-BMD in the IVW (beta-estimate: 0.396, 95% CI: -0.128 to 0.866, SE:0.253, *P*-value = .340) or weighted-median analyses (beta-estimate: 0.150 95% CI: -0.385 to 0.684, SE: 0.273, *P*-value = .583, Table 3). Low heterogeneity was seen for the association between education and LS-BMD (Q-value = 9.143, P-heterogeneity = 0.1657, I^2^ = 34%). MR estimates of education on FA-BMD and LS-BMD were shown in Figures 2 and 3, separately.

### 3.3. Causal effect of education on HE-BMD

**Figure 4.**
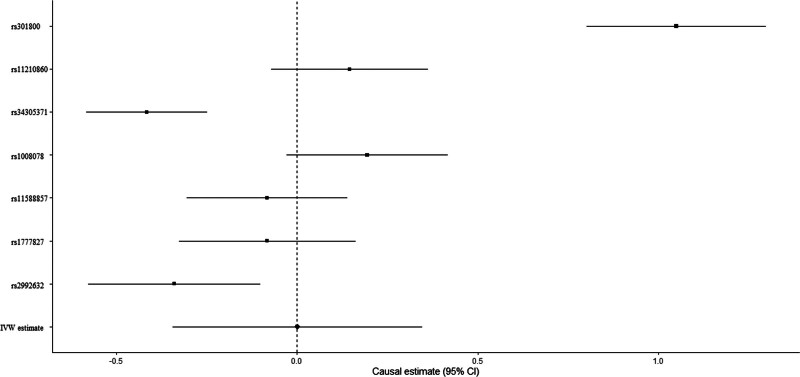
Mendelian randomization association between education and HE-BMD. HE-BMD = heel bone mineral density.

Education demonstrated no obvious association with HE-BMD according to the IVW (beta-estimate: 0.001, 95% CI: −0.344 to 0.346, SE: 0.176, *P*-value = .995) or weighted-median analyses (beta-estimate: −0.083, 95% CI: −0.234 to 0.069, SE: 0.077, *P*-value = .285, Table 3). There was significant heterogeneity between them (Q-value = 105.854, P-heterogeneity < .0001, *I*^2^ = 94%). Figure 4 revealed the MR association between education and HE-BMD.

### 3.4. Evaluation of assumptions and sensitivity analyses

The strength of the genetic instruments was denoted by the F-statistic, and they were all ≥ 10 for all variants, indicating that no weak instrument variables remained (Table 1). There was little evidence of directional pleiotropy for all models (MR-Egger intercept *P*-values > .05, Table 3). The estimates from the weighted-median approach for SNP instrument were all consistent with those of IVW models (Table 3).

## 4. Discussion

In this MR analysis, high level of educational attainment was significantly associated with improved FN-BMD, and this positive result was confirmed by weighted-median analysis. There was no causal effect of educational attainment on other sites of BMD. These findings indicated that the increase in education level may benefit to lower the incidence of osteoporosis.

Previous studies reported some conflicting results between educational attainment and osteoporosis. A cross-sectional data from the Third National Health and Nutrition Examination Survey revealed the positive relationship between education and BMD among Black and White postmenopausal women.^[32]^ Ho reported that high level of education was independently associated with improved BMD and low prevalence of osteoporosis among postmenopausal Chinese women.^[33]^ However, no significant association was observed between education level and osteoporosis in another cross-sectional study of Taiwan.^[34]^ Lauderdale reported a favorable association between high educational status and BMD among premenopausal women from the United States but not among postmenopausal immigrant women from Vietnam, Cambodia and Laos.^[12,15]^

These inconsistent results may be derived from the methodological limitations (i.e., confounding, reverse causation and measurement error) of a traditional observational study.^[35]^ The design of randomized controlled trial (RCT) is the gold standard to study causal inference, but it is not feasible to explore the association between education and osteoporosis because of the long latency period between exposure and outcome, as well as the unethical approaches of limiting education in childhood. The two-sample MR study is widely used to evaluate causal inferences between risk factors and disease outcomes by using genetic variants as instrumental variables.^[36]^

Our study included the large GWAS meta-analysis regarding educational attainment among 293,723 individuals, the large GWAS meta-analysis associated with FA-BMD, FN-BMD, and LS-BMD among 53,236 people, and GWAS summary data regarding HE-BMD among 426,824 individuals. The casual association between high education level and improved FN-BMD was revealed based on the results and sensitivity analyses of this two-sample MR study.

Several mechanisms may explain the contribution of high education level to increase BMD. Many risk factors are associated with osteoporosis, including hormonal factors, poor diet, use of certain drugs, cigarette smoking, low physical activity and BMI, low intake of calcium, and vitamin D.^[37]^ These factors are prevalent in populations with low education level and socioeconomic position.^[13,38]^ In addition, populations with no formal education tend to become older easily and to have high number of pregnancies, high duration of veil wearing, low daily calcium intake and physical activity level than others.^[11]^ Better-educated individuals may tend to have better health knowledge and behavior in developed countries. Increasing affluence and education can help improve the nutrition and healthy lifestyles.^[39,40]^ For example, increasing evidences showed that peak bone mass among Iranian population was lower than European and American populations.^[41]^ One cross-sectional study involved 706 women aged 50 to 75 years old, and revealed that the prevalence of osteoporosis among low educated women was approximately 5 times more than high educated women.^[37]^ These results were consistent with the findings in western countries.^[42,43]^

The association between education level and fracture risk was rarely reported. Perez found that the robust association between low education level and high risk of hip fracture in Spanish and Turkish women.^[44]^ Wilson documented that patients without high school diploma had more than a 2-fold risk of hip fracture than those with high school diploma.^[45]^ The positive association between educational levels and risk of hip fracture was confirmed among ambulatory non-Hispanic White men.^[46]^

This two-sample MR study aims to investigate the causality between education and BMD, which is the closest approximation to RCT and allows the random allocation based on the genotype. This study design can prevent some limitations of conventional observational studies, including reverse causation and potential confounding factors. The large sample sizes of included studies and instrumental variables robustly associated with education (F statistics ≥ 10) are used. The intercepts for the MR-Egger analysis suggest that all observed causal associations are not affected by directional pleiotropy.

Several limitations also should be taken into consideration. Firstly, all the included participants are of European origin, and more studies should be conducted to confirm whether our results are useful to other populations. Secondly, this MR study reveals the causal effect of education on FN-BMD, but null association is observed between education and other sites of BMD. The factors to result in this inconsistency remain elusive. Thirdly, it is not feasible to perform the MR analysis based on different age stratums because of the limitation of GWAS summary statistics.

## 5. Conclusion

This two-sample MR confirmed that high educational attainment had an importantly causal role in improving FN-BMD.

## Acknowledgments

The authors acknowledged the GEnetic Factors for OSteoporosis Consortium and the UK Biobank for contributing the data used in this work.

## Author contributions

**Conceptualization:** Mingqi Sun, Xiaojun Chen.

**Data curation:** Xiaoqing Mou, Mingqi Sun, Xiaojun Chen.

**Formal analysis:** Xiaoqing Mou, Mingqi Sun, Xiaojun Chen.

**Funding acquisition:** Xiaojun Chen.

**Investigation:** Xiaoqing Mou, Mingqi Sun, Xiaojun Chen.

**Methodology:** Xiaoqing Mou, Mingqi Sun, Xiaojun Chen.

**Project administration:** Xiaoqing Mou, Mingqi Sun, Xiaojun Chen.

**Resources:** Xiaoqing Mou, Mingqi Sun.

**Software:** Xiaoqing Mou, Mingqi Sun.

**Supervision:** Xiaojun Chen.

**Validation:** Xiaoqing Mou, Xiaojun Chen.

**Visualization:** Xiaojun Chen.

**Writing – original draft:** Xiaojun Chen.

**Writing – review & editing:** Xiaojun Chen.
